# Editorial: Fluoride exposure, dental fluorosis, and health

**DOI:** 10.3389/froh.2023.1256495

**Published:** 2023-08-03

**Authors:** Rogelio González-González, Ronell Bologna-Molina, Nelly Molina-Frechero

**Affiliations:** ^1^ Research Department, School of Dentistry, Juarez University of the Durango State, Durango, Mexico; ^2^ Molecular Pathology Area, Diagnostics in Pathology and Oral Medicine School of Dentistry, University of the Republic, Montevideo, Uruguay; ^3^ Division of Biological and Health Sciences, Autonomous Metropolitan University Xochimilco, Mexico City, Mexico

**Keywords:** fluorosis, high intake, intoxication, bone alterations, organ damage, dental fluorosis

**Editorial on the Research Topic** Fluoride exposure, dental fluorosis, and health

Adequate absorption of fluoride, a micronutrient, is related to the strengthening of dental organs and bone tissue; however, excess consumption of fluoride causes damage at the dental, bone, and systemic levels. Drinking water is a rich source of fluoride, whose concentration levels should not exceed 1.5 mg/L (1). Nevertheless, in various countries, the concentration is higher than the levels recommended by the World Health Organization (1), causing high intake toxicity. Toxicity not only depends on the intake but is also related to the exposure time, diet, and nutritional status of the individual, as well as the altitude of the location. The purpose of the Research Topic “Fluoride Exposure, Dental Fluorosis, and Health,” was to evaluate fluoride exposure in relation to health damage. This study includes four articles that assess the risks of high fluoride intake from various perspectives.

The stomach and small intestine absorb fluoride at a systemic level quickly, distributing it throughout the body. Excess absorption causes alterations in dental tissue, bone tissue, organs, and systems (Tefera et al.). When constantly consumed in large amounts for a long time, fluoride can cause chronic disease. The first sign of chronic toxicity is observed through changes in the dental organs, which show a creamy-white surface, white and yellow spots, lines or striations, and porosity. In more severe injuries, the tooth enamel becomes weak, brittle, prone to fractures or ruptures, and has significant areas of wear and tear.

Dental fluorosis is one of the first clinical signs of fluorosis and can be associated with skeletal and systemic fluorosis, affecting the bones, joints, organs, and entire systems (2). According to a study by Levy et al., dental fluorosis becomes less noticeable during adolescence and early adulthood. Thus, the levels of toxicity at the systemic level do not decrease even though the dental signs are less severe. Hence, further studies on fluoride concentrations in urine with the use of biomarkers—such as nails or hair—are suggested in these populations (3).

Tefera et al. have described skeletal fluorosis in Ethiopian school-age children (6–13 years of age), in whom the primary manifestation observed was the inability to stretch and cross their arms to touch the back of their head, revealing valuable clinical characteristics in the evaluation of bone damage by fluorosis. The authors also indicated that these manifestations are related to a diet with excessive fluoride consumption and low calcium intake. Calcium is thus an important mineral for reducing the absorption of fluoride in bones and teeth.

Saad et al. in a systematic review, evaluated the fluoride content in dental hygiene products, observing a high daily intake in children <4 years of age and adults who did not follow good dental care practices. According to this study, it is advisable to use consumable dental hygiene products cautiously; for example, toothpaste should not be swallowed, the mouth must be rinsed with water after brushing teeth, and an adequate amount of toothpaste must be used; these may be important factors in decreasing daily fluoride intake. Adhering to these suggestions in populations with high exposure will help to control the daily consumption of fluoride through consumer products and thus contribute to the reduction of fluoride intake.

Irigoyen–Camacho et al. evaluated the association between dental fluorosis and fluoride intake in children with low socioeconomic status. The study was carried out in Oaxaca, Mexico, where fluoride concentrations in groundwater are >0.7 ppm; here, the children with poor nutrition presented with high levels of fluoride excretion in their urine. Thus, malnourished or undernourished children may experience negative effects from fluoride at doses that may be safe for well-nourished children. These characteristics are possibly observed because calcium consumption levels are lower in children who are not well nourished.

Therefore, in communities with high levels of fluoride consumption and poor nutrition, preventive measures must be undertaken in addition to the proper use of dental care products containing fluoride (toothpaste, varnishes, or other dental care products). These are important factors in controlling the daily intake of fluoride in such communities (Saad et al.).
